# Linkage of nanosecond protein motion with enzymatic methyl transfer by nicotinamide N-methyltransferase

**DOI:** 10.3906/biy-2101-54

**Published:** 2021-06-23

**Authors:** Yahui JING, Yiting CHENG, Fangya LI, Yuping LI, Fan LIU, Jianyu ZHANG

**Affiliations:** 1 School of Pharmaceutical Science and Technology, Tianjin University, Tianjin China; 2 Binzhou Institute for Food and Drug Control, Binzhou, Shandong China

**Keywords:** Protein dynamics, nicotinamide N-methyltransferase, methyl transfer, time-resolved fluorescence

## Abstract

Nicotinamide N-methyltransferase (NNMT), a key cytoplasmic protein in the human body, is accountable to catalyze the nicotinamide (NCA) N^1^-methylation through S-adenosyl-L-methionine (SAM) as a methyl donor, which has been linked to many diseases. Although extensive studies have concerned about the biological aspect, the detailed mechanism study of the enzyme function, especially in the part of protein dynamics is lacking. Here, wild-type nicotinamide N-methyltransferase together with the mutation at position 20 with Y20F, Y20G, and free tryptophan were carried out to explore the connection between protein dynamics and catalysis using time-resolved fluorescence lifetimes. The results show that wild-type nicotinamide N-methyltransferase prefers to adapt a less flexible protein conformation to achieve enzyme catalysis.

## 1. Introduction

The secret of how the enzyme can achieve such amazing catalytic capabilities has not been fully revealed yet. From protein movement (Bruice, 2002; Hammes et al., 2011; Da et al., 2014), electrostatic preorganization effect (Warshel, 1998; Henzlerwildman and Kern, 2007; Hammes-Schiffer and Sharon, 2013) to the near attack conformation (NAC) (Warshel and Levitt, 1976), different theories with controversial issues to uncover the origin of enzyme catalytic ability have been derived. Despite these disputes, the development from the lock and key theory to the induced-fit and conformational selection theory shows us some common ideas: proteins are not rigid and static, but dynamic and flexible. Therefore, we should explore not only the protein function and average structure, but also the relative probabilities of conformational states. However, whether protein movement in nanosecond timescale could be related to protein functions is still under debate and has aroused our great interest. Advanced techniques for characterizing dynamic properties including nuclear magnetic resonance (NMR) (Nodet and Abergel, 2007), fluorescence spectroscopy (Lakowicz, 1999), molecular dynamics simulation (Karplus and Kuriyan, 2005), and X-ray crystallography (Srajer et al., 1996). Among them, fluorescence spectroscopy can be a responsible approach for investigating small-scale protein dynamics in the nanosecond range. Here, nicotinamide N-methyltransferase and its mutants were selected and combed with enzyme activity and protein fluorescence measurements to explore the relationship between protein dynamics and protein function.

**Scheme 1:**
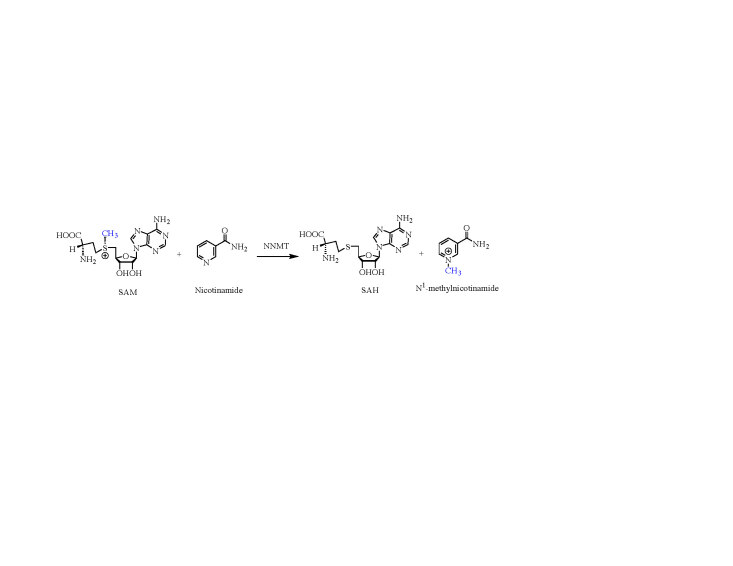
The methylation process of nicotinamide catalyzed by nicotinamide N-methyltransferase (NNMT).

Nicotinamide N-methyltransferase (NNMT) is accountable to catalyze the N^1^-methylation of nicotinamide (NCA) (Scheme 1) and makes a critical difference in liver detoxification and energy metabolism (Liscombe et al., 2012). Deviant expression of NNMT is connected with diverse diseases, containing various types of tumors (Lim et al., 2006; Ulanovskaya et al., 2013; Sartini et al., 2015), obesity, diabetes (Kraus et al., 2014) and Parkinson’s disease (Parsons et al., 2003). NNMT exists in the liver predominately, but also expressed in some other organs (Aksoy et al., 1994; Kraus et al., 2014). A rapid equilibrium ordered mechanism has been shown to be used by NNMT (Loring and Thompson, 2018), with SAM binding first, followed by nicotinamide. 

**Figure 1:**
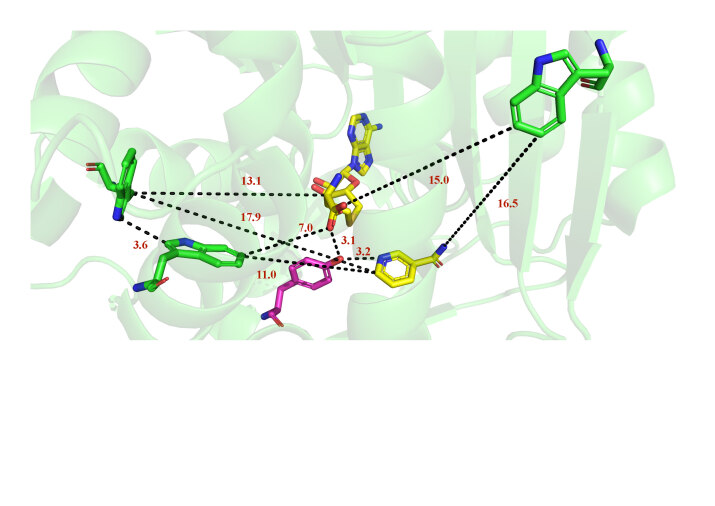
Crystal structure of NNMT (PDB: 3ROD) with tryptophan residues Trp97, Trp107 and Trp234, Tyr20, S-adenosyl-Lhomocysteine (SAH) and nicotinamide (NCA). (Oxygen: red; nitrogen: blue; carbon in green for all tryptophans; purple for tyrosine 20 and yellow for SAH and nicotinamide.)

From the crystal structure of human NNMT (PDB: 3ROD), three tryptophan residues, Trp97, Trp107, and Trp234 can be seen (Figure 1). Due to the low tryptophan content, the explanation of fluorescence data is not that complicated as there is no intertryptophan interactions (Chattopadhyay and Haldar, 2014). Trp97 and Trp107 located in α5 and α6 helices of the central domain respectively. However, Trp234 belongs to β6 antiparallel strands from the C-terminal domain. In addition, the crystal structure shows that Y20, as an active site residue, has a side chain hydroxyl group interact both to the nicotinamide ring and the carboxylate of S-adenosyl-L-homocysteine (SAH) (Figure 1). It is shown that Y20 plays a vital role in NNMT function (Peng et al., 2011). Site-specific mutagenesis has been applied to generate Y20F and Y20G, using as probes for their effect on changes in catalytic efficiency and protein dynamic properties. Results reveal that as the catalytic ability of the protein reduces, the fluorescence lifetime decreases, indicating that mutation at position Tyrosine 20 can change the conformational balance of protein, resulting in changes in conformational frequency, thereby affecting the function of the protein.

## 2. Materials and methods

### 2.1. Mutation and protein purification

To purify NNMT and its mutants, we performed as previously with some modificaitons (Peng et al., 2011; Van Haren et al., 2016; Nemmara et al., 2018). Wild type NNMT plasmid was synthesized by TSINGKE biological technology. The mutants Y20F and Y20G were constructed by PCR using the Fast Site-Directed Mutagenesis Kit (TIANGEN, Beijing, China). For Y20F, the forward primer and reverse complement are (5’-TTTAACCCTCGGGATTTCCTAGAAAAATATTAC-3’) and (3’-AAATTGGGAGCCCTAAAGGATCTTTTTATAATG-5’), respectively. For Y20G, the forward primer and reverse complement are (5’-TTTAACCCTCGGGATGGCCTAGAAAAATATTAC-3’) and (3’-AAATTGGGAGCCCTACCGGATCTTTTTATAATG-5’), respectively. The vector was transformed into *Escherichia coli* BL21 (DE3) competent cells and then cultivated in 37 °C LB medium. The cells were induced with 1 mM IPTG (isopropyl β-D-1-thiogalactopyranoside) when the OD_600_ was reach to around 0.6. Wet cells were obtained by centrifuge at 5000 × g for 20 min (4 °C). Wet cells were resuspended in the lysis buffer at 4 °C and then sonicated, PMSF (phenylmethylsulfonyl fluoride, 1 mM) was added in advance. After lysis, supernatant was obtained by centrifuging at 20,000 × g for 17 min (4 ºC). In order to eliminate the SAM generated in the expression, the clear supernatant was incubated at 37 °C (1 h) with 1 μM GNMT (glycine N-methyltransferases) and 20 mM glycine. Then the reaction mixture was cooled in ice and applied to a Ni-NTA metal affinity column (Superflow Cartridges, Qiagen, Hilden, Gemany ) that had been preequilibrated. The column was washed by the wash buffer for at least 30 column sizes of the column. Elution buffer was used to collect NNMT. SDS-PAGE was applied to check the molecular weight and purity of the proteins. The pure NNMT was obtained and dialyzed against dialysis buffer. Finally, the protein was concentrated and stored it at –80 ºC for further use. Bradford assay was used to calculate protein concentration. 

### 2.2. Steady-state kinetic measurements

Enzyme catalytic activity for NNMT and its mutants (Y20F and Y20G) was measured by the fluorescence intensity produced by the reaction of N-methyl nicotinamide with 2-acetylbenzofuran. The concentration ranges from 0.05 mM to 0.25 mM for nicotinamide in NNMT wild type system. For Y20F and Y20G system, the concentration was varied from 0.08–3.6 mM to 1–48 mM for nicotinamide, respectively. The purified enzyme and nicotinamide were preincubated at 37 ºC in 50 mM Tris-HCl (pH = 8.6) and then SAM was introduced to initiate the reaction. Samples of 80 μL were taken at a sequence of different time points and an equal volume of ethanol was introduced to quench the reaction. The quenched mixture was mixed with 200 mM 2-acetylbenzofuran (20 μL) and an equal volume of 1 M KOH then placed for 30 min (4 °C). After that, 200 μL formic acid was introduced for another 30 min (room temperature). 200 μL of the samples was taken for determination. The excitation and emission wavelengths were set as 418 nm and 458 nm respectively to measure the fluorescence intensity.

### 2.3. Model reaction

Nicotinamide (4 mL, 0.2 M) and methyl iodide (2 mL, 8 mM) were added to 14 mL of anhydrous ethanol and stirred in the dark for 1 h (78 °C). The reaction was quenched with an equal amount of 30 mM ammonia. The resulting products were quantified using fluorescence as mentioned above.

### 2.4. Steady-state fluorescence determination

The measurements were performed as previously with some modifications (Liu and Zhang, 2020). The emission spectrum of NNMT was measured with a fluorescence spectrometer FLS980-STM (Edinburgh Instruments), with Xe1-300 and red PMT-400 as the excitation source and the emission detector, respectively. Excitation wavelength was set to 295 nm to eliminate the contribution of tyrosine and phenylalanine to the fluorescence. The emission spectrum ranges from 300 nm to 600 nm with an interval of 5 nm. The steady-state fluorescence was determined in 50 mM Tris-HCl (pH 8.6) containing DTT (1,4-dithiothreitol, 1 mM). Fluorescence spectra were collected with a 3 μM enzyme or free tryptophan at 10 °C. The protein emission spectrums were corrected by deducting the buffer background fluorescence. 

### 2.5. Fluorescence lifetime determination

The fluorescence lifetimes were determined by the usage of time-correlated single-photon counting technique (TCSPC), and FLS980-STM was employed as excitation source (Meadows et al., 2014; Liu and Zhang, 2020). The decay plots were collected at 335 nm as 295 nm is the excitation wavelength. When both the emission and excitation wavelength were set to 295 nm, the instrument response function (IRF) could be determined. Collecting 10,000 peak counts for all measurements to get the fluorescence decay curves. Each data set was collected in 1024 channels and measured over a time range of 50 ns. The actual fluorescence decay curves of proteins were obtained after subtracting the buffer background in the F980 workstation. Reconvolution fit was conducted to obtain the optimal fitting result. The fluorescence decay was fitted to Eq. 1, which is the sum of discrete exponentials.

*R* (t,λ)= Σ^n^_i=1 _α_i_(λ)exp[-t/τ_i _(λ)] (1)

Where n is defined as the number of exponents needed for fitting the attenuation data, α_i_(λ) is the amplitude and τ_i_(λ) is the fluorescence lifetime for the *i* th component at a certain wavelength.

## 3. Results and discussion

**Figure 2:**
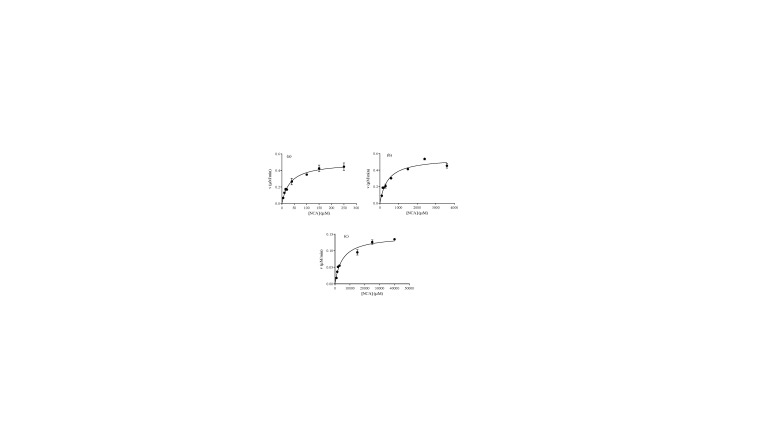
Michaelis–Menten plot of NNMT WT, Y20F and Y20G. (a) NNMT WT, (b) Y20F, (c) Y20G.

The crystal structure of the complex of human NNMT with substrate nicotinamide and SAH shows that Tyr20, which is located in the α2 helix of the N-terminal domain, resides right behind the sulfur of bond SAH. The phenolic hydroxyl group of Tyr20 not only interacts with the oxygen of the carboxylate of SAH, but also with the nitrogen of the aromatic ring of nicotinamide (Figure 1). Moreover, previous studies have indicated that Tyr20 side chain is essential for the NNMT function (Peng et al., 2011). Therefore, theTyr20 site-directed mutants were generated as controls to compare the impact of the mutation on protein activity with the wild-type protein. As the residue 20 is changed from Tyr to Phe or Gly, both turnover number (k_cat_) and the catalytical efficiency (k_cat_/K_m_) are reduced (Table 1, Figure 2). The turnover number when cofactor and substrate are in a saturated state indicates a 2.5-fold decrease in Y20F compared to WT and more significantly compromised (230-fold decrease) in the smaller side chain Y20G. The catalytic efficiency is quite sensitive to the mutagenesis, showing around a 30-fold decrease for nicotinamide of Y20F (from 2488 ± 309 M^–1^S^–1^ for WT to 87 ± 22 M^–1^S^–1^ for Y20F), indicates that the substrate binding is up to the electrostatic interaction produced by the phenolic hydroxyl group. Moreover, as the residue 20 was changed from Tyrosine to hydrophobic glycine, the enzyme activity showed an even more dramatic reduction (3.5 × 10^4^-fold reduction for nicotinamide). This impact on changes in enzyme activity reveals that the catalytic process also depends upon the package and orientation of the benzene ring to the substrate. However, in a previous study (Peng et al., 2011), the catalytic efficiency of Y20A showing around 36-fold reduction for nicotinamide, indicating that the change of catalytic efficiency after tyrosine mutation to glycine is more sensitive than that after tyrosine mutation to alanine. In order to further understand the function of the enzyme, we have carried out the model study of the methyl transfer in solution. The model reaction uses methyl iodine to react with nicotinamide to simulate the methyl transfer reaction without enzyme, the rate is 2.6 ± 0.1 × 10^–5^ min–1 at 78 °C. This chemical methylation efficiency is much lower than that of any enzyme-catalyzed system, where a 1.9 × 10^5^-fold difference could be observed compared to the reaction catalyzed by wild-type NNMT. 

**Table 1:** Kinetic parameters for nicotinamide catalyzed by NNMT and its mutants at 37 °C .

NNMT	kcat (min–1)	Km (nicotinamide) (μM)	kcat/Km (M–1S–1)
WT	5.0 ± 0.2	33 ± 4	2488 ± 309
Y20F	2.0 ± 0.1	388 ± 92	87 ± 22
Y20G	0.022 ± 0.002	4958 ± 1411	0.074 ± 0.022
Model reaction	2.6 ± 0.1 × 10–5 a	─	─

**Figure 3:**
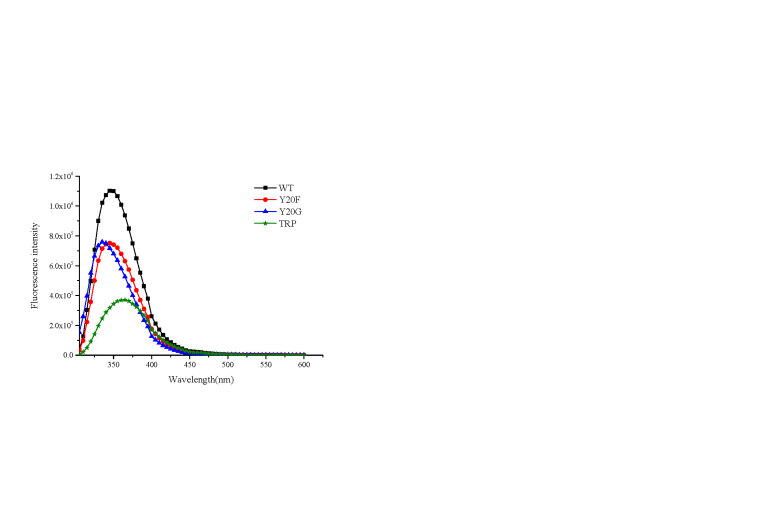
The steady-state fluorescence emission spectra of NNMT WT, Y20F, Y20G, and free tryptophan at 10  C ([C] = 3μM for all samples). The excitation wavelength is 295 nm.

The steady-state fluorescence emission spectra for wild-type NNMT together with Y20F, Y20G, and free tryptophan were measured as shown in Figure 3. Steady-state fluorescence reflects changes in protein conformations and the maximum emission spectrum of protein fluorescence could give information about the mean exposure of tryptophan to the aqueous solution (James et al., 1985; Sopkova et al., 1994). The results show that both wild-type NNMT and Y20F have the maximum emission wavelength at 345 nm, and as the side chain at position 20 is changed from tyrosine to glycine, a blue shift with maximum emission wavelength at 335 nm was observed. This suggested that the mutation from polar tyrosine to hydrophobic glycine changed the protein’s conformation and reduced the aqueous phase accessibility of the tryptophan residues. The free tryptophan solution shows a fluorescence emission band at 365 nm. Compared with NNMT-WT and its mutants, this red shift indicates that the average environment of free tryptophan is more polar than that of enzymes. Furthermore, the fluorescence intensity of wild-type NNMT and its mutants are higher than that of the same concentration of free tryptophan, suggesting that the polarity of the excited state of free tryptophan is stronger than the ground state. The excited fluorescent molecules tend to interact with polar solvents (or polar environment), with the change of electron distribution of solvent molecules. This will affect the ground state and excited state energy levels of fluorescent molecules with reduced excited state energy, resulting in lower fluorescence intensity of free tryptophan in polar water environment (Lakowicz, 1999).

**Figure 4:**
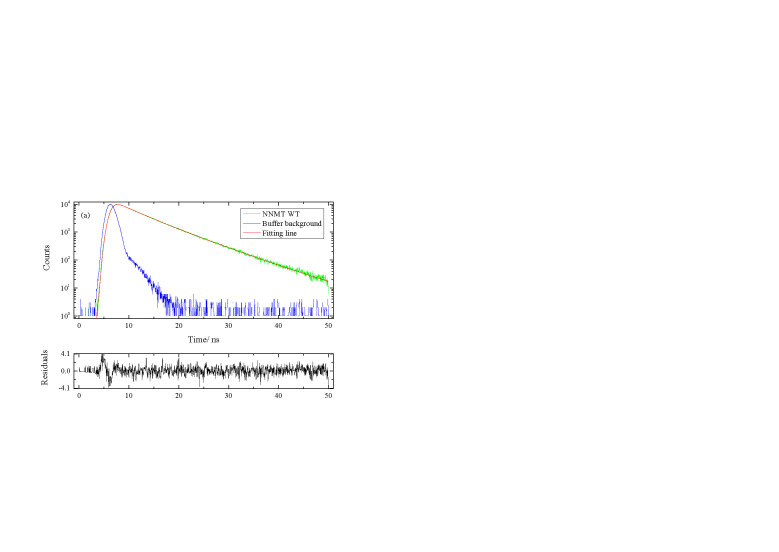

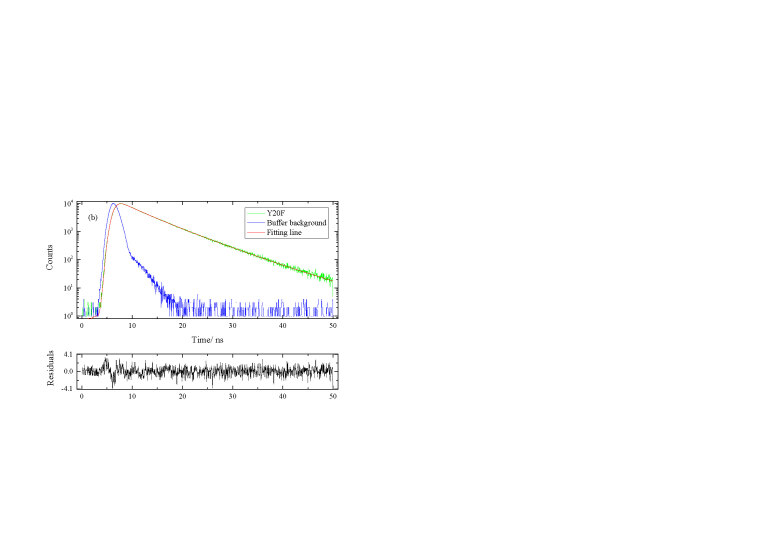

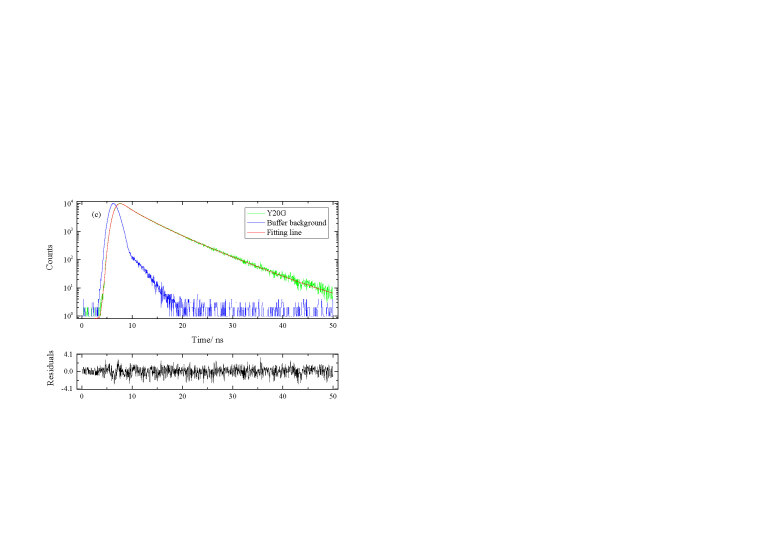

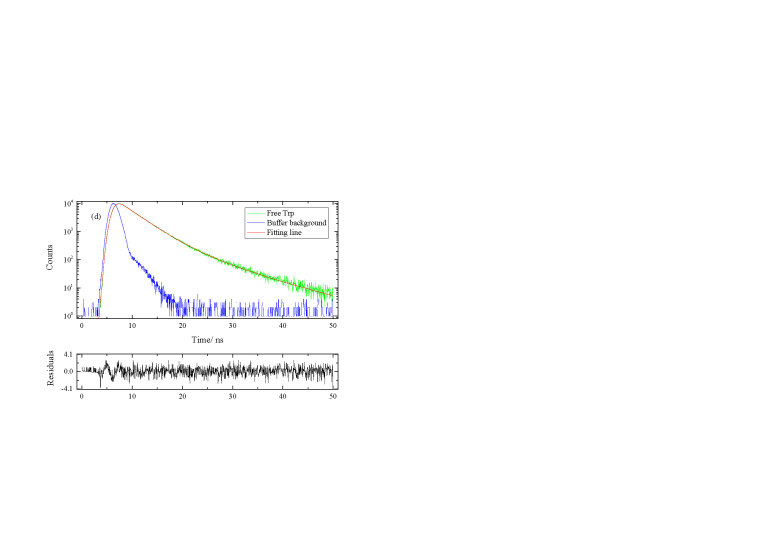
Fluorescence lifetime decays and residue errors at 335 nm for NNMT WT, Y20F, Y20G and free tryptophan at 10  C. (a) NNMT WT, (b) Y20F, (c) Y20G, (d) Free Trp. The χ2 is 1.17 (NNMT WT), 1.16 (Y20F), 0.99 (Y20G) and 1.07 (Free Trp).

**Figure 5:**
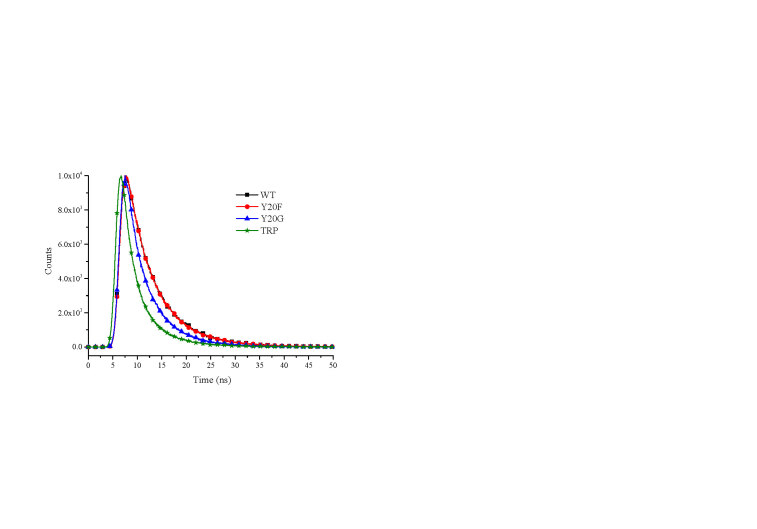
Fluorescence lifetime decays at 335 nm for NNMT WT, Y20F, Y20G, and tryptophan at 10  C.

For the sake of obtaining the detailed information about protein conformation, the lifetime decay of NNMT and its mutants was measured by nanosecond time-resolved fluorescence spectroscopy (Figures 4 and 5). The lifetime decay data fitted best to a three-exponential model as shown in Table 2. From Table 2, it was interesting to find that with the mutation of residue 20 from Tyr to Phe or Gly, the average fluorescence lifetime <τ> of the protein reduced from 5.93 ns to 5.85 ns or 4.45 ns, respectively. Furthermore, the average lifetime <τ> for tryptophan solution is the lowest with the value of 3.69 ns. This trend of lifetime decrease upon mutation can also be observed as shown in Figure 5. The specific information about the contribution of different protein conformations to the overall fluorescence lifetime can be obtained from Table 2. 

**Table 2:** Representative fluorescence decay parameters for NNMT WT, Y20F, Y20G, and free tryptophan. All data are obtained with an emission wavelength of 335 nm at 10 °C.

NNMT	α1	τ1(ns)	α2	τ2(ns)	α3	τ3(ns)	<τ>a	χ2b
WT	0.21	3.21	0.64	6.19	0.15	8.68	5.93	1.17
Y20F	0.10	2.57	0.43	4.83	0.47	7.48	5.85	1.16
Y20G	0.10	0.77	0.41	3.19	0.49	6.30	4.45	0.99
Tryptophanc	0.04	1.52	0.82	3.25	0.14	6.94	3.69	1.07

The fluorescence lifetime in the ps region is thought to be caused by effective tryptophan quenching, while the ns timescale fluorescence lifetime may result from the non-quenched conformational changes of protein (Henzlerwildman and Kern, 2007). The increasing trend of fluorescence decay rate from wild-type NNMT to free tryptophan suggests a mutation-induced local protein conformational change. Specifically, the fluorescence lifetime is related to the flexibility of the protein: the longer lifetime, the less flexible of protein (Alcala, 1994). It can be seen that proteins become more flexible after a series of mutations, with the least flexible for wild-type protein and most flexible for free tryptophan.

Combining the enzyme activity and lifetime decay study, an interesting phenomenon was observed: as the catalytic ability of the protein reduces, the fluorescence lifetime decreases with increasing protein flexibility. The enzyme itself has many conformations, with one of the intrinsic conformations help the catalysis occurs (Eisenmesser et al., 2005). In other words, the intrinsic dynamic properties of enzymes are essential for catalytic function. The mutation at position 20 changes the protein conformational balance, resulting in the changes in the frequency of conformations and the fluorescence lifetime. It is also suggested that the flexibility of the protein reflected by the fluorescence decay is closely related to the catalytic ability of the protein. As we all know that the process of catalysis requires an accurate balance between flexibility and stability. The data obtained indicate that compared with the Y20 mutants with lower activity, the catalytic movement of the substrate binding domain at position 20 in wild-type NNMT prefers a less flexible structure.

## 4. Conclusion

The present study reports the linkage of the nanosecond protein dynamics and catalytic efficiency of NNMT using fluorescence spectroscopy and steady-state kinetic measurements. The results revealed a change in protein conformational balance by mutations, which could lead to the corresponding change of enzyme function: wild-type NNMT prefers the less flexible conformation to achieve high catalytic power. A more detailed and comprehensive study about how different conformational changes affect the function of the protein is required. This work provides a forward step to fully establish the connection between protein function and protein conformation motion, which is believed to the key point for the design of artificial enzyme and protein engineering. 
